# End-to-End Encrypted Message Distribution System for the Internet of Things Based on Conditional Proxy Re-Encryption

**DOI:** 10.3390/s24020438

**Published:** 2024-01-10

**Authors:** Shi Lin, Li Cui, Niu Ke

**Affiliations:** 1 School of Cryptographic Engineering, Engineering University of PAP, Xi’an 710000, China; slshilin@126.com; 2 School of Information and Communication, National University of Defense Technology, Wuhan 430000, China; lc_licui17@nudt.edu.cn

**Keywords:** internet of things, end-to-end encryption, conditional proxy re-encryption, message broker, HiveMQ

## Abstract

In light of the existing security vulnerabilities within IoT publish–subscribe systems, our study introduces an improved end-to-end encryption approach using conditional proxy re-encryption. This method not only overcomes limitations associated with the reliance on a trusted authority and the challenge of reliably revoking users in previous proxy re-encryption frameworks, but also strengthens data privacy against potential collusion between the broker and subscribers. Through our innovative encryption protocol, unauthorized re-encryption by brokers is effectively prevented, enhancing secure communication between publisher and subscriber. Implemented on HiveMQ, an open-source MQTT platform, our prototype system demonstrates significant enhancements. Comparison to the state-of-the-art end-to-end encryption work, encryption overhead of our scheme is comparable to it, and the decryption cost is approximately half of it. Moreover, our solution significantly improves overall security without compromising the asynchronous communication and decentralized authorization foundational to the publish–subscribe model.

## 1. Introduction

To realize data communication among a large number of entities, large-scale Internet of Things (IoT) system generally uses the publish–subscribe (pub/sub) paradigm for data distribution. The most commonly used protocols which work in the pub/sub paradigm are Message Queuing Telemetry Transport (MQTT) [1], Advanced Message Queuing Protocol (AMQP) [2], etc. The subscribers can subscribe to a “message topic”, and publisher can publish messages to the “message topic”. All subscribers will receive the publisher’s message through the routing of the message broker between the publisher and subscribers. The pub/sub paradigm decouples the senders and receivers in time and space. They do not need to be directly connected or online simultaneously. It is more flexible, efficient, and scalable than the point-to-point data exchange mode. A typical pub/sub-based IoT system includes three types of components: IoT devices, a message broker, and a user management application. The device and the management application serve as the publisher and subscriber, respectively. The device publishes the data collected by its sensor to a specific topic, and the authorized user of the device subscribes to that topic through the application. The message broker in the middle routes the data to all the authorized users.

At present, Transport Layer Security (TLS) [3,4] is widely used in the industry to protect the data between the client (publisher or subscriber) and the message broker. The broker decrypts the ciphertext from the publisher to obtain the plaintext, encrypts the plaintext again with the key negotiated with each subscriber, and then forwards the corresponding ciphertext to the subscriber. However, the broker can obtain all the data generated by the client and has complete control over the user’s data [5]. Furthermore, the message broker maintained by IoT manufacturers is not completely trusted. Recent research shows that no-security policies are implemented by a large number of accessible message brokers, which allows anyone to receive data or inject messages [6]. Some researchers investigated a specific traffic monitoring system and discovered that the MQTT message broker was disclosing the traffic flow conditions in a specific area of Mexico City [7]. Even if the message broker deploys a security policy, users need to totally trust the broker. Currently, the message broker in pub/sub-based IoT system are deployed either by IoT device manufacturers based on existing commercial message servers (such as EMQX [8], HiveMQ [9] or Solace [10]), or based on the IoT cloud platform provided by third-party cloud computing manufacturers (such as Alibaba cloud [11], Amazon AWS cloud [12]). The message broker is established and maintained by the IoT device manufacturer or the IoT cloud platform [13]. These manufacturers are not entirely reliable. If the administrator operates incorrectly or is bribed by a spy, or if the manufacturer is for profit purposes, user data are likely to be abused or shared with unauthorized entities, which poses a threat to the security of user data.

In addition, at present, most pub/sub-based IoT systems are constructed based on MQTT protocol, which is not designed for hostile environments. Jia Yan et al. [14] found that the MQTT protocol has serious defects. The platform using this protocol can enable adversaries to steal users’ private information and forge users’ device status.

In order to prevent the threats brought by malicious message brokers, PICADOR [15] uses proxy re-encryption (PRE [16]) technology to provide end-to-end encryption from publishers to subscribers. In PRE, given a re-encryption key to the semi-trusted proxy, the proxy can convert the ciphertext encrypted with the public key of user A into the ciphertext encrypted with the public key of user B without obtaining any plaintext information from the ciphertext. The proxy re-encryption process can be described as: $E\left(pk_{A},m\right)\overset{rk_{A\to B}}{\to }E\left(pk_{B},m\right)$ ]; here, $rk_{A\to B}$ is the re-encryption key from A to B.

In PICADOR, the publisher encrypts its message with its public key. The broker re-encrypts the published message using the re-encryption key of each subscriber and then sends the corresponding ciphertext to each subscriber. The subscriber can decrypt the ciphertext with their private key. PICADOR needs a trusted authority to generate the re-encryption key for each subscriber according to the publisher’s private key and each subscriber’s public key. When revoking the authorization of a subscriber, the simplest way is that the broker no longer re-encrypts messages for the revoked subscribers. However, the broker is not entirely trusted. If it is compromised and still re-encrypts for the revoked user, then the revoked user can still receive the latest message. Therefore, depending on the broker for user revocation is not completely reliable. If we do not rely on the broker to revoke users, then we can only change the public–private key pair of the publisher once revocation is required and regenerate the re-encryption key for all the remaining authorized users, which would result in frequent changes to the public–private key pair of the publisher. The long-term public–private key of the user is usually used for authentication either, both between users and between users and brokers. If the user’s public–private key pair changes frequently, then there will be inconveniences encountered during authentication.

The reason of the problem in PICADOR is that traditional proxy re-encryption allows the proxy to convert all ciphertexts without restriction [17]. As long as the proxy possesses the re-encryption key from A to B ( $rk_{A\to B}$ ), the proxy can convert all ciphertexts encrypted with the public key of A into ciphertexts which could be decrypted using the private key of B. This all-or-nothing feature is not suitable for applications that need fine-grained authorization of decryption capability. Based on this, Weng et al. proposed the concept of Conditional Proxy Re-encryption (CPRE) [18], which allows for the conditional conversion of ciphertext. In CPRE, when generating ciphertext using user A’s public key, a condition value is introduced at the same time, and the re-encryption key from A to B are also related to a condition value. Only when the condition value used for generating the ciphertext is equal to the condition value related to the re-encryption key, the proxy can convert the ciphertext encrypted with the public key of A into the ciphertext encrypted with the public key of B. A can prevent the proxy from performing unauthorized re-encryption by controlling the change of condition value [17]. The process of conditional proxy re-encryption can be described as: $E\left(pk_{A},m,\omega \right)\overset{rk_{A\overset{\omega^{\prime }}{\to }B}}{\to }E\left(pk_{B},m\right)\left(\omega =\omega^{\prime }\right)$ .

Because CPRE has significant advantages over PRE in fine-grained authorization, this paper introduces CPRE for the first time to realize end-to-end encryption in pub/sub-based IoT system to prevent broker from performing unauthorized re-encryption. We investigate a large number of existing conditional proxy re-encryption schemes. According to the principles of low computing and communication overhead and high security, the conditional proxy re-encryption algorithm proposed by Weng et al. in 2009 [19] is selected in our system.

In our system, the publisher uses its private key, conditional value, and subscriber’s public key to generate the conditional re-encryption key for each subscriber, and sends the conditional re-encryption key to the broker. When publishing a message, the publisher encrypts the message with its public key and condition value and sends the ciphertext to the broker. The broker uses the re-encryption key of each subscriber to re-encrypt the message and then sends the re-encrypted ciphertext to the corresponding subscriber. Finally, the subscriber can obtain the plaintext by decrypting the message with their private key. When a subscriber is revoked, the publisher updates the condition value and generates new condition re-encryption keys for each remaining subscriber. Specifically, the main contributions of our system are as follows:

(1) The conditional proxy re-encryption (CPRE) algorithm is introduced to solve the end-to-end encryption problem in the pub/sub-based IoT system. the re-encryption key is associated with a condition value. By changing the condition value, the publisher can ensure that the proxy can not perform unauthorized re-encryption, thereby achieving reliable revocation of subscribers.
(2) By using an open-source MQTT message server, HiveMQ, we implement a prototype end-to-end encryption system for a pub/sub-based IoT system based on CPRE, and further enhance the system’s performance through hybrid encryption and hash chain. Moreover, the performance of the system is tested, which shows that our system is not only easy to implement on existing commercial message servers, but also has high performance.

## 2. Related Works

### 2.1. End-to-End Encryption in IoT

At present, a large number of scholars have studied the end-to-end security in pub/sub-based systems [20,21]. This section survey the current research status of end-to-end encryption schemes according to the technology used.

**The scheme based on a trusted message broker:** Jia Yan [14] proposed MOUCON to solve access control issues in MQTT, the MQTT broker in MOUCON is responsible for verifying each client’s access to the message. Clients must fully trust the broker and cannot resolve the security threat to user data caused by an untrusted broker.

**The scheme based on the trusted key server:** Markus et al. [5] propose an end-to-end security scheme for Cyber–Physical Systems (CPS). The scheme relies on trusted key servers to distribute topic keys for publishers and subscribers. The key server stores the global authorization information of the system and the encryption key. If the key server is compromised by the adversary, all the account information, authorization information, and the encryption keys of the system will be exposed.

**The scheme based on identity-based encryption (IBE):** JEDI [20] realizes the end-to-end encryption between devices and users in IoT, facilitates asynchronous communication, and supports decentralized authorization for the key. The scheme does not require any modifications to the message broker, which is convenient for deployment. However, this method uses the identity-based encryption algorithm with wildcards (WKD-IBE) [21], the algorithm has a high computational complexity, making it unsuitable for resource-constrained IoT devices.

**The scheme based on secret sharing:** Sana Belguith et al. [22] proposed an efficient and revocable secure publish–subscribe system. The system divided the broker into three parts to handle the functions of topic matching, routing, and message sending separately. As long as the adversary does not simultaneously break all three brokers, the solution remains secure. However, this solution requires the customization of a special message broker, which is inconvenient to deploy.

**The scheme based on special hardware:** Segarra et al. [23] restrict the broker to run only in a trusted execution environment (TEE) [24], thus ensuring that the broker functions as intended by the deployer. However, the installation and deployment of TEE requires professional management, as well as its maintenance, which results in high costs. In addition, there are security attacks against the current mainstream TEE [25], so TEE still has some security risks.

**The scheme based on proxy re-encryption:** PICADOR [15] implements end-to-end encryption between publishers and subscribers using proxy re-encryption. As can be inferred from the above analysis, this scheme depends on a trusted authorization center to generate re-encryption keys and relies on the broker to revoke users. However, this approach has the drawback of unreliable revocation.

### 2.2. Conditional Proxy Re-Encryption Schemes

Mambo and Okamoto [26] first introduced the concept of decryption capability authorization, which has higher performance than the method of decrypting and then encrypting the ciphertext. In 1998, Blaze, Bleuner, and Strauss formally introduced the concept of proxy re-encryption (PRE) [16], and since then, lots of research has been carried out around PRE. PRE allows a semi-trusted agent to transform the decryption capability of a ciphertext without obtaining any valid information about the ciphertext, and is widely used in encrypted email transmission, secure distributed file systems, and encrypted spam filtering, etc. PRE can be categorized according to different criteria. Based on the direction of re-encryption, PRE can be categorized into one-way PRE and two-way PRE. Additionally, based on the number of re-encryptions allowed, it can be categorized into single-hop PRE and multi-hop PRE.

Traditional proxy re-encryption is unable to provide fine-grained authorization of decryption capabilities. In response, Weng et al. introduced the concept of conditional proxy re-encryption (CPRE) [18] and developed the first CPRE scheme. The re-encryption key of the scheme consists of two parts: the re-encryption key and the conditional key. However, the scheme only considered the security of the second ciphertext layer and not the first ciphertext layer. Weng [27] pointed out that the scheme described in the literature [18] is vulnerable to Chosen-Ciphertext Attacks (CCAs). Weng [27] redefined a more stringent security model for CPRE and proposed a new efficient CPRE scheme. Both Shao [28] and Liang [29] proposed CCA-secure identity-based CPRE under the DBDH (Decisional Bilinear Diffie–Hellman Problem) assumption. However, the literature [30] points out that the scheme given by Liang [29] is insecure.

Fang et al. [31] proposed an anonymous CPRE scheme that enables keyword search. Subsequently, Jae Woo Seo et al. [32] proposed a type-based privacy requirement engineering (Type-based PRE) scheme, where “type” refers to a keyword that is equivalent to “condition” in CPRE. Thus, this scheme is essentially similar to the CPRE scheme, which achieves fine-grained authorization of user decryption capabilities. Son et al. [33] proposed a CPRE for big data sharing on cloud platforms by outsourcing the re-encryption key generation and decryption to the servers. Qiu et al. [19] and Liang et al. [34] proposed CCA-secured CPREs, respectively. Ge et al. [35] proposed an identity-based CPRE scheme. The authors proposed an identity-based CPRE scheme that enables contingent gate computation on conditionals. Hu Xiong et al. [36] introduced a unidirectional multi-hop identity-based CPRE scheme that facilitates flexible and efficient data authorization in cloud computing environments and demonstrated its security in the standard model. Arinjita et al. [37] presented a conditional proxy re-encryption scheme that does not require pairwise operations. The scheme is not reliant on the bilinear pair operation for construction and has lower computational overhead. However, in this scheme, if the receiver conspires with the agent, the agent can compute the sender’s private key as long as it possesses two conditional re-encryption keys. Therefore, the scheme proposed by Arinjita et al. [37] is not able to resist the conspiracy attack between the agent and the receiver.

The following compares various CPRE (Conditional Proxy Re-encryption) schemes, and the results are presented in Table 1 and Table 2. The schemes proposed in the literature [18,29,37] exhibit security issues and are therefore not included in the comparison. Let |G| and |GT| denote the bit lengths of elements in groups *G* and GT , respectively. $|Z_{p}|$ represents the bit length of elements in the prime field $|Z_{p}|$ . |m| stands for the bit length of the plaintext. $t_{p}$ and $t_{e}$ represent the time required for a single bilinear pairing operation and a single exponentiation operation, respectively. |σ| represents the bit length of the signature output by a strongly unforgeable one-time signature algorithm. svk is the length of the verification key for the strongly unforgeable one-time signature algorithm. $t_{v}$ is the time taken to verify a strongly unforgeable one-time signature. *t* represents the size of the access tree, and *w* denotes the size of attributes.

In the practical implementation of the CPRE algorithm, the re-encryption algorithm is executed by a semi-trusted agent, which is generally deployed on servers or clouds with more abundant resources. In contrast, the encryption and decryption operations are typically performed by IoT devices or personal handheld electronic devices with significantly smaller computational resources than the agent. Therefore, the overall principle in selecting CPRE algorithms is to choose the scheme with lower computational and communication overhead. When computational and communication overheads are comparable, the scheme with lower encryption and decryption overheads is chosen. In terms of security, the schemes in the table are all provably CCA secure in either the standard model or the stochastic prediction model. Although the schemes proven to be secure under the standard model are theoretically more reliable, in this paper, we choose the schemes proven to be secure under the stochastic prediction model. This is because the security of such security schemes depends only on the hash function itself, and no practical attack has yet occurred that can compromise a practical cryptographic algorithm proven to be secure under the stochastic prediction model (excluding some carefully constructed counterexamples by humans [38]). In addition, these security schemes are computationally more efficient and have a wider range of applications.

Based on the aforementioned principles, this paper utilizes the conditional proxy re-encryption algorithm proposed by Weng et al. [27] in 2009 to achieve the encryption of the publish–subscribe system of this paper from the publisher side to the subscriber side.

## 3. Preliminaries

**Figure 1:**
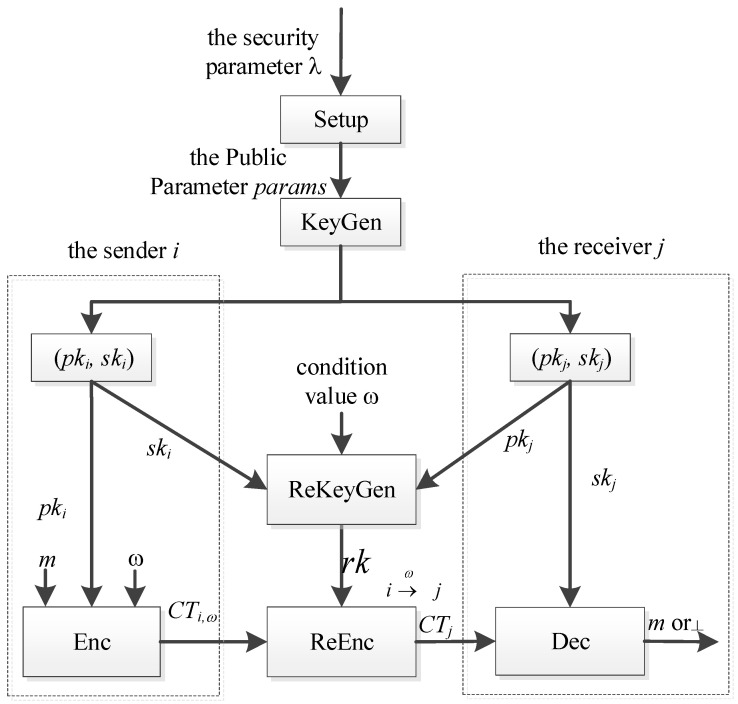
Workflow of CPRE.

The conditional proxy re-encryption CPRE scheme includes the following algorithms, and its workflow is given in Figure 1:

$Setup\left(\lambda^{k}\right)$ : Input the security parameter $\lambda^{k}$ , and the algorithm outputs the public parameter params .

$KeyGen\left(\lambda^{k}\right)$ : Each entity uses this randomized key generation algorithm to generate a public–private key pair $\left(pk_{i},sk_{i}\right)$ .

$ReKeyGen\left(sk_{i},\omega ,pk_{j}\right)$ : Input the private key $sk_{i}$ of the sender, the condition value ω and the public key $pk_{j}$ of the receiver, the re-encryption key generation algorithm outputs the re-encryption key $rk_{i\overset{\omega }{\to }j}$ from sender *i* to receiver *j*.

$Enc_{1}\left(pk_{i},m\right)$ : Input the public key $pk_{i}$ of the sender and plaintext *m*, the first layer encryption algorithm outputs the first layer ciphertext $CT_{i}$ . The ciphertext cannot be re-encrypted.

$Enc_{2}\left(pk_{i},m,\omega \right)$ : Input the public key $pk_{i}$ of the sender, the plaintext *m* and the condition value ω , the second-layer encryption algorithm will output the second-layer ciphertext $CT_{i,\omega }$ . This ciphertext can be re-encrypted using an appropriate re-encryption key into a first-layer ciphertext for different recipients.

$ReEnc\left(CT_{i,\omega },rk_{i\overset{\omega }{\to }j}\right)$ : Input the second-layer ciphertext $CT_{i,\omega }$ , the re-encryption key $rk_{i\overset{\omega }{\to }j}$ , the proxy runs the re-encryption algorithm to output the first-layer ciphertext $CT_{j}$ .

$Dec_{1}\left(CT_{j},sk_{j}\right)$ : Input the first-layer ciphertext $CT_{j}$ and private key $sk_{j}$ , the first-layer decryption algorithm outputs plaintext *m* or error symbol ⊥.

$Dec_{2}\left(CT_{i,\omega },sk_{i}\right)$ : Input the second-layer ciphertext $CT_{i,\omega }$ and private key $sk_{i}$ , the second-layer decryption algorithm outputs the plaintext message *m* or error symbol ⊥.

By introducing a conditional value into the generation of the re-encryption key and the second layer encryption algorithm, the conditional proxy re-encryption algorithm ensures that the proxy cannot perform unauthorized re-encryption.

## 4. End-to-End Encryption System Based on CPRE

### 4.1. System Framework

Our CPRE-based end-to-end encryption system consists of three types of entities: IoT devices (referred to as the sender), message broker, and multiple authorized users (referred to as the receiver).

**Figure 2:**
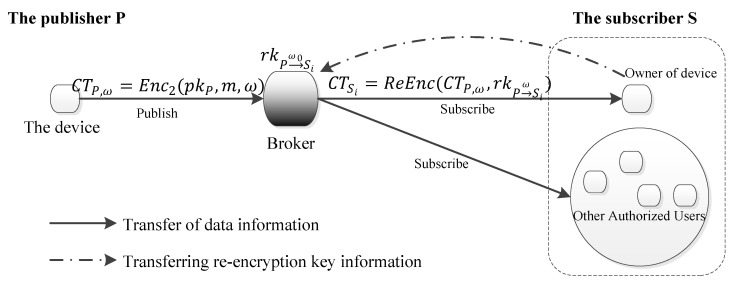
The framework of IoT end-to-end encryption system.

Our system utilizes the conditional proxy re-encryption algorithm proposed by Weng et al. [19] to achieve end-to-end encryption from the publisher to the subscribers in pub/sub-based IoT system. The specific algorithm design can be found in the literature [19]. In our system, the device owner generates a re-encryption key for each authorized user and send the re-encryption key to the Broker. The device acts as the sender and encrypts the message with its public key and a condition value. The broker re-encrypts for each subscriber using the corresponding re-encryption key. All authorized users can decrypt the ciphertext using their private key. The framework of our system is shown in Figure 2.

CPRE differs from PRE in that its re-encryption key $rk_{P\overset{\omega }{\to }S_{i}}$ and the second-layer ciphertext $CT_{P,\omega }$ are both associated with a condition value ω . If the authorization for certain users need to be revoked, the device owner just need to generate a new condition value $\omega^{\prime }$ and send to the device. Then, the device owner generates a new re-encryption key $rk_{P\overset{\omega^{\prime }}{\to }S_{i}}$ for each remaining recipient with the device’s private key $sk_{P}$ , the new condition value $\omega^{\prime }$ , and the public keys of the remaining authorized users $pk_{S_{i}}$ . The owner no longer generates re-encryption keys for the revoked users. The device uses the new condition value $\omega^{\prime }$ to generate the second layer ciphertext $CT_{P,\omega^{\prime }}$ . The broker re-encrypts with the new re-encryption key $CT_{P,\omega^{\prime }}$ for the remaining authorized users, so that the remaining legitimate users can decrypt the message correctly. Since the re-encryption key of the revoked users is not updated, it is still associated with the previous condition value ω , and the new ciphertext corresponds to the updated condition value $\omega^{\prime }$ . Even if the broker is compromised and still re-encrypts the message for the revoked users—as the condition value in the re-encryption key and second layer ciphertext are not equal—the revoked user cannot decrypt the re-encrypted ciphertext correctly with their private key.

### 4.2. System Workflow

Specifically, the workflow of our CPRE-based end-to-end encryption scheme includes the following steps.

#### 4.2.1. User Registration

When a user wants to utilize our system for device management or monitoring, he must complete the user registration process through the client application. The algorithm $KeyGen\left(1^{k}\right)$ of CPRE is integrated into the application, allowing the user to generate their public and private key pairs $\left(pk_{S_{i}},sk_{S_{i}}\right)$ . The user keeps their private key $sk_{S_{i}}$ secret.

#### 4.2.2. Device Registration

When a user purchases a new IoT device, the user is the owner of the device and is responsible for device registration and authorization control. Typically, a newly purchased IoT device starts its life cycle with “device discovery” [3]. In this stage, the device owner requests to add a device through the client APP, and the APP establishes a local connection with the device to complete the device registration and the binding of the device and its owner. We assumes that during the “device registration” phase, the device interacts with the client APP of the device owner through a local connection, exchanging basic information and performing mutual authentication. During the device registration process, the device owner utilizes the built-in key generation algorithm $KeyGen\left(1^{k}\right)$ of the APP to generate public and private key pairs $\left(pk_{P},sk_{P}\right)$ for the device. Additionally, the owner generates a random initial condition value $\omega_{0}$ , and transfers the initial condition value $\omega_{0}$ and device’s public–private key pair $\left(pk_{P},sk_{P}\right)$ to the device through the “Local Connection” established in registration phase. The device owner also keeps the private key $sk_{P}$ of the device secretly, which is used to generate the conditional re-encryption key for other authorized users.

#### 4.2.3. Authorization Phase

When the device owner wants to authorize the access rights of the device to other users, the device owner uses the private key $sk_{p}$ of the device, the condition value $\omega_{0}$ , and the public key $pk_{S_{i}}$ of each authorized user to generate a conditional re-encryption key $rk_{P\overset{\omega_{0}}{\to }S_{i}}$ for each authorized user, which will be sent to the broker of the publish–subscribe system.

#### 4.2.4. Message Transmission Stage

The device runs the CPRE encryption algorithm, encrypts the collected information with its public key $pk_{P}$ and condition value $\omega_{0}$ and obtain the ciphertext $CT_{P,\omega_{0}}=Enc_{2}\left(pk_{p},m,\omega_{0}\right)$ , which will be sent to the broker. Then, the broker re-encrypts the ciphertext according to the conditional re-encryption key $rk_{P\overset{\omega_{0}}{\to }S_{i}}$ of each authorized user and sends the re-encrypted ciphertext $CT_{S_{i}}=ReEnc\left(CT_{P,\omega_{0}},rk_{P\overset{\omega_{0}}{\to }S_{i}}\right)$ to the corresponding authorized user. Finally, each authorized user uses their private key $sk_{S_{i}}$ to decrypt the ciphertext $CT_{S_{i}}$ and obtains the plaintext $m=Dec_{1}\left(CT_{S_{i}},sk_{S_{i}}\right)$ .

#### 4.2.5. Revocation Phase

When the device owner needs to revoke the access permission of one user, first, the device owner randomly selects a new condition value $\omega_{1}$ ; then, the owner generates a new conditional re-encryption key for each remaining authorized user with the user’s public key $pk_{S_{j}}$ , the device’s private key of $sk_{P}$ , and the new condition value $\omega_{1}$ . Furthermore, the updated re-encryption keys are sent to the broker. Finally, the device owner encrypts the new condition value with the public key of the device and sends the ciphertext to the device.

The device decrypts with its private key $sk_{P}$ to obtain the new condition value $\omega_{1}$ , it updates the condition value to the new one. Similarly, when the broker receives the new conditional re-encryption key $rk_{P\overset{\omega_{1}}{\to }S_{j}}$ distributed by the device owner, the conditional re-encryption key will also be updated from $rk_{P\overset{\omega_{0}}{\to }S_{i}}$ to $rk_{P\overset{\omega_{1}}{\to }S_{j}}$ . Then, the device encrypts the message with the new condition value, and the broker re-encrypts with the new conditional re-encryption key.

### 4.3. System Optimization

#### 4.3.1. Hybrid Encryption

Most IoT devices are low-power devices with limited resources, so we use hybrid encryption to further reduce device-side overhead. Before encrypting the collected messages, the device first selects a random symmetric key *k*, encrypts the key with CPRE and sends to the broker. Each authorized subscriber can decrypt the re-encrypted ciphertext with their private key, thereby obtaining the same symmetric key *k*. Since then, subsequent communications between the device and each subscriber can be encrypted using the symmetric key *k*.

#### 4.3.2. Hash Chain

Whenever the set of authorized users changes (such as new users join or old users are revoked), if CPRE is used to generate new conditional re-encryption keys for all remaining authorized users, when the authorized user set changes frequently, generating and transmitting conditional re-encryption keys imposes significant overhead on the device owner. Therefore, when a new user joins, a symmetric key is distributed to the new user by means of the hash chain [39]. The CPRE algorithm is used only when the user is revoked.

Specifically, the process of distributing a symmetric key to a new user by using a hash chain is as follows: Assume that the symmetric key shared between the device and each subscribing user is *k* before the new user joins. Before a new user joins, the device owner uses *k* as the input of the one-way trapdoor function to obtain a new session key $k_{1}=Hash\left(k\right)$ , and sends the new session key to the new joined user. At the same time, the device owner broadcasts the key update command, so that the device and other legitimate users can also update their shared key *k* to $k_{1}$ through the one-way trapdoor function. Then, the consistency of the shared session key between the device and all its authorized users can be ensured.

### 4.4. System Analysis

Below, we analyze our CPRE-based end-to-end encryption system in IoT.

#### 4.4.1. Satisfy Confidentiality

When a new user is authorized to join, the session key is updated through the hash chain. Based on the unidirectionality of the hash function, the new user cannot deduce the session key *k* before joining by using the session key $k_{1}$ .

When the user is revoked, the system uses CPRE to update the session key. The device owner randomly selects a new condition value, and generates new conditional re-encryption keys for the remaining users, but no longer generates new conditional re-encryption keys for revoked users. Therefore, when the device uses CPRE encryption to transmit a randomly selected new session key, the new condition value is used, and the broker also uses the new conditional re-encryption key for re-encryption, so that the remaining legitimate users can use their own private key to decrypt and obtain the new session key. The conditional re-encryption key of the revoked user is also related to the old condition value, even if the broker colludes with the revoked user, still re-encrypts with the old conditional re-encryption key. As the condition value in the cipertext of the device is not equal to the condition value in the revoked user’s re-encryption key, so the revoked user cannot decrypt or obtain the new session key. Based on the above analysis, the revoked user cannot obtain a new session key even if he colludes with the broker. In addition, our scheme is constructed based on the CPRE algorithm, and the broker can only use the re-encryption key to convert the ciphertext, and cannot obtain any plaintext message of the ciphertext based on the security proof of CPRE in [19].

#### 4.4.2. Support Asynchronous Communication

When a new user joins, the session key is updated through the hash chain, and the offline user does not affect the session key update process between the online user and the device. When the user is revoked, the update of the conditional re-encryption key involves the device owner. As long as the device owner is online, the broker can obtain the latest conditional re-encryption key, and the device can obtain the latest conditional value, offline users do not affect the process. In short, offline users do not affect the key update process of online users, and our scheme supports asynchronous communication.

#### 4.4.3. Support Decentralized Authorization

In our scheme, the authorization and revocation of device access rights are only controlled by its owner and do not rely on trusted third parties. If a device owner is compromised by an adversary, only the security of the owner and its managed devices will be affected, and the security of other devices and users will not be affected.

#### 4.4.4. Support the Decoupling of Publishers and Subscribers

Our scheme is designed based on the CPRE scheme. The device uses its public key to encrypt the data. The device does not need to encrypt for each authorized user, and does not need to care about which users subscribed to its data. The broker completes the conversion and distribution of the ciphertext sent by the device. Therefore, the publisher (ie, device) and subscriber (ie, user) in our scheme are decoupled, and their relationship is controlled and managed by the device owner.

## 5. Prototype Implementation and Performance Analysis

This section implements the above-mentioned IoT end-to-end encryption system and tests the system’s performance.

### 5.1. Implementation of the Prototype System

We implement each module based on the Java language. Our prototype system includes three types of entities: the publisher, multiple subscribers, and the message broker which can perform re-encryption operations. The implementation of the CPRE algorithm [19] is based on the JPBC (the Java Pairing-Based Cryptography Library) [40]. The establishment of the message broker is based on the open-source MQTT server HiveMQ [9], which supports customized function extensions. The development of the publisher and the subscriber is based on the Eclipse Paho Java Client library [41]. The prototype system runs on a laptop configured with an Intel Core i7-5600U 2.6GHZ CPU and 8G RAM (Intel, Santa Clara, CA, USA). The computer operating system is Windows 7. The development environment of the publisher, subscriber, and Broker is IntelliJ IDEA 2018.1.6. The functions of the publisher and subscriber are simulated using a Java console program.

#### 5.1.1. CPRE Implementation

The CPRE scheme used in our system is constructed based on the symmetric bilinear group. Therefore, we use the configuration file “a.properties” provided by the JPBC library to generate a type A symmetric bilinear group based on a prime-order elliptic curve $y^{2}=x^{3}+x\mathrm{mod}p\left(p\equiv 3\mathrm{mod}4\right)$ , while the base field size is 512 bits, and the embedding degree is 2.

#### 5.1.2. Implementation of Message Broker

The construction of Broker is based on the open-source MQTT server HiveMQ Community Edition [42], which provides the SDK (HiveMQ Extension SDK) [43] that supports extension development. Through this, users can develop custom business logic to extend the functions of HiveMQ with the SDK, such as intercepting or controlling MQTT messages, integrating other services, statistics, and adding fine-grained security solutions. We use the core part of HiveMQ to implement message routing and forwarding, implements message re-encryption in a customized extension, and re-encrypts the message payload for each subscriber based on the re-encryption key of each subscriber.

HiveMQ includes multiple types of Interceptors, which provide convenience for intercepting and modifying MQTT messages in extensions. Our customized extension is implemented through HiveMQ Interceptors. The goal of our customized extension is to re-encrypt the payload of the message according to the subscriber’s re-encryption key after the message is routed, but before the message is forwarded to the subscriber. Therefore, our customized extension is implemented using the **Publish Outbound Interceptor** in HiveMQ, which allows the extension to intercept the PUBLISH message after the broker is routed, and allows different modifications to the payload corresponding to each subscriber.

#### 5.1.3. Implementation of the Client

The implementation of the publisher and the subscriber is relatively easy, which is based on the Eclipse Paho Java Client library and the JPBC library is added to support the encryption and decryption of the published and received messages based on CPRE.

### 5.2. Performance Analysis

This section tests the performance of each module of our system based on the prototype system built in the previous section.

#### 5.2.1. Overhead of Distributing Session Keys Using CPRE

**Figure 3:**
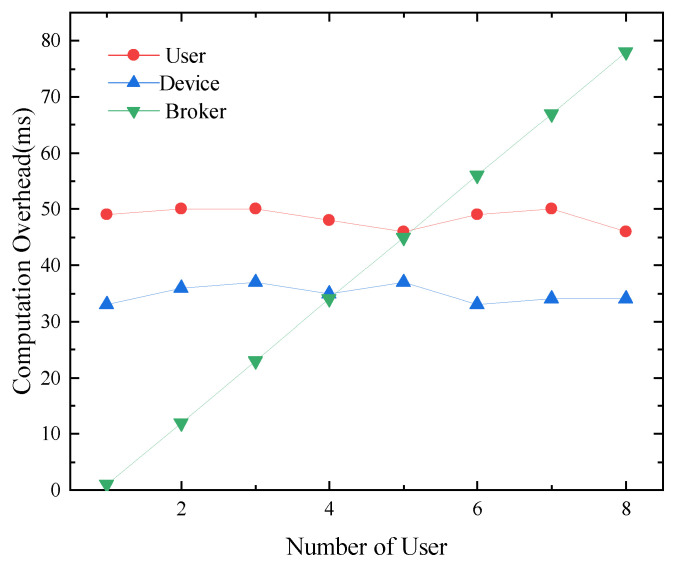
Computation overhead of each module to distribute session key based on CPRE.

When the device receives a new condition value, it will randomly generate a new session key. The key will be encrypted with the device’s public key and the new condition value, and then ciphertext will be sent to the Broker. The broker re-encrypts the ciphertext for each subscriber and sends the re-encrypted ciphertext to each subscriber. Each subscriber decrypts the re-encrypted ciphertext with its private key, and obtains the session key. When the device uses CPRE to transmit an AES (Advanced Encryption Standard) [44] key (128 bits), the computational overhead of each module (device, broker, and subscriber) in our system is shown in Figure 3.

The computation overhead of the device is approximately 46 ms. The overhead of the subscriber to is approximately 35 ms. The processing time required by the broker increases linearly with the number of users. As shown in Figure 3, for each additional user, the processing time of the Broker increases by approximately 9 ms. The Broker is generally deployed on a server or cloud platform with rich resources, which can easily cope with this overhead increase. The computation overhead of the device and users does not change with the number of users, making it suitable for IoT devices with limited resources.

#### 5.2.2. Overhead of Secure Communication

After the device and the subscriber have established a shared symmetric key, the communication between the device and the subscribers are encrypted by the symmetric encryption algorithm AES. We compare the computation overhead of the publisher and subscriber when they use AES for message transmission than when they transmit in plaintext.

**Figure 4:**
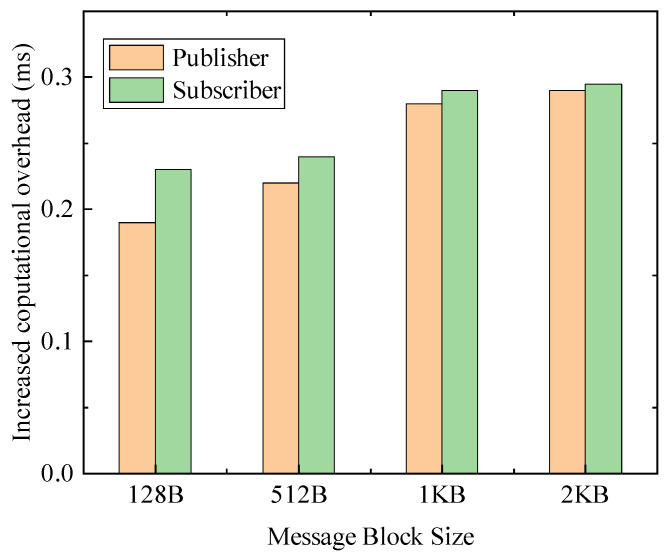
Increased computation overhead for encrypted transfers.

For multiple message sizes (128 B, 512 B, 1 KB, 2 KB), Figure 4 shows that encrypted transmission between the publisher and the subscriber incurs an increased overhead of approximately 0.2–0.3 ms per message. Therefore, the use of symmetric keys for secure transmission brings only a slightly increase in computation cost.

#### 5.2.3. Comparison with Related Schemes

Table 3 compares the current end-to-end encryption schemes in IoT in terms of security, whether it supports decentralized authorization, whether it is convenient to deploy, and performance.

- Security: The message brokers in most IoT systems are not completely trusted. In a scheme that completely relies on the message broker, the broker can obtain all the information of the user, which does not meet the confidentiality requirements.
- Support decentralized authorization: If relying on a third-party trusted key server, the authorization and revocation of device access rights must be completed through the key server, and decentralized authorization is not supported. PICADOR relies on a trusted authority to generate re-encryption keys for all users of the system and does not support decentralized authorization too.
- Easy to deploy: Reference [22] divides the functions of a broker into multiple ones, and new brokers need to be customized to meet the corresponding functions. Reference [39] needs to install special hardware, which is difficult to deploy.
- Performance: The schemes that rely on trusted brokers do not meet the confidentiality requirements, and the literature [22,23] is difficult to deploy. Therefore, these schemes are not re-implemented on our experimental platform. Ref. [5] relies on a trusted key server, the scheme uses symmetric key to establish session key, so [5] distributes symmetric keys faster than our scheme. However, in the secure communication stage, the overhead of using symmetric keys to encrypt and transmit messages is equal to our scheme. Ref. [20] implements the scheme of PICADOR, whose performance is comparable to [20]. In order to have a fair comparison with [20], we re-implement the WKD-IBE algorithm in [20] using our crypto library. In [20], the encryption algorithm takes almost 42 ms to encrypt 128 bits of data, and the decryption algorithm takes about 62 ms to decrypt and obtain the plaintext (the decryption time contains the time to generate a decryption key for the encrypted pattern and time to decrypt the ciphertext. When testing the computation overhead, we use a pattern of 20 attributes representing the URI and the last six attributes representing the time.). The encryption overhead of our scheme is comparable to that presented in [20], while the decryption cost is approximately half that presented in [20].

Above all, some existing schemes rely on the trustworthiness of the broker, and their security assumptions are strong, which cannot meet the confidentiality requirements; some schemes rely on a third-party trusted server for authorization and revocation, and do not support decentralized authorization; some solutions need a customized broker or rely on special hardware, which is inconvenient to deploy. Ref. [20] is a solution with good security and deployability. However, it relies on the more complex WKD-IBE algorithm, and its performance is lower compared to our proposed solution.

## 6. Conclusions

In the publish–subscribe-based IoT system, the communication between devices and users is not one-to-one direct communication, but one-to-many asynchronous communication and forwarded by the broker located in the middle. Currently, TLS is commonly employed to safeguard the security of data transmission between the device and the broker. However, the broker has the ability to access the plaintext of all messages, rendering this method ineffective in preventing the security and privacy risks posed by untrustworthy brokers to device data. We present and implement a new end-to-end encryption system based on conditional proxy re-encryption. Theoretical analysis and experimental results demonstrate that our proposed scheme is not only theoretically safe, but also highly practical, feasible, and efficient in practice. However, our system cannot efficiently revoke users, which will form the focus of our future research.

## Figures and Tables

**Table 1:** Comparison of the computation overhead of each CPRE algorithm.

Scheme	Re-Encryption Key Generation	Encrypt	Re-Encrypt	Decrypt
[27]	$4t_{e}$	$3t_{e}+t_{p}$	$3t_{p}$	$2t_{e}+t_{p}$
[28]	$5t_{e}+t_{p}$	$8t_{e}+t_{p}$	$6t_{p}$	$4t_{e}+8t_{p}$
[31]	$2t_{e}$	$t_{e}$ +5 $t_{p}+t_{s}$	$2t_{e}+t_{p}+t_{v}$	$t_{e}+t_{p}$
[32]	$t_{e}$	$5t_{e}+t_{p}$	$5t_{e}$ +2 $t_{p}$	$2t_{e}+4t_{p}$
[33]	$4t_{e}$	$3t_{e}$ +2 $t_{p}$	$t_{e}$ +2 $t_{p}$	$3t_{e}$ +2 $t_{p}$
[19]	$3t_{e}$	$4t_{e}+t_{p}$	$4t_{p}$	$2t_{e}+t_{p}$
[34]	$9t_{e}+t_{p}$	$5t_{e}+t_{p}$	$6t_{e}$ +7 $t_{p}$	$5t_{e}$ +12 $t_{p}$
[35]	$8t_{e}+t_{p}+t_{s}$	$6t_{e}+t_{p}+t_{s}$	$\left(9+2w+t\right)t_{p}+t_{v}$	$t_{e}+8t_{p}+t_{v}$
[36]	$6t_{e}$	$6t_{e}+2t_{p}$	$t_{e}+4t_{p}+t_{v}$	$t_{e}+3t_{p}+t_{v}$

**Table 2:** Comparison of the communication overhead of each CPRE algorithm.

Scheme	Initial Ciphertext	Re-Encryption Key	Ciphertext after Re-Encryption
[27]	$2|G|+|G_{T}\left|+|m\right|$	2|G|	$2|G|+|G_{T}\left|+|m\right|$
[28]	$4|G|+|G_{T}\left|+\right|Z_{p}|$	$2|G|+2|G_{T}$ |+2| $Z_{p}$ |	$4|G|+|G_{T}\left|+|m\right|$
[31]	$\left|G|+3\right|G_{T}\left|+svk+|\sigma \right|$	$2|G|+2|Z_{p}$ |	$2|G_{T}\left|+svk+|m\right|$
[32]	$2|G|+|G_{T}\left|+svk+|\sigma \right|$	$\left|G|+\right|Z_{p}$ |	$4|G|+|G_{T}\left|+svk+|\sigma \right|$
[33]	$\left|G|+2\right|G_{T}$ |	2|G|	$2|G|+2|G_{T}$ |
[19]	2|G|+|m|	2|G|	$|G_{T}\left|+|m\right|$
[34]	$4|G|+|G_{T}\left|+svk+|\sigma \right|$	$6|G|+|G_{T}\left|+svk+|\sigma \right|$	$3|G|+|G_{T}\left|+svk+|\sigma |+|m\right|$
[35]	$\left(w+4\right)|G|+|G_{T}\left|+w+|\sigma |+|m\right|$	$6|G|+|G_{T}|+|\sigma |+t$	$\left(w+6\right)|G|+2|G_{T}\left|+2|\sigma |+2|m\right|$
[36]	$3|G|+2|G_{T}\left|+svk+|\sigma \right|$	2 $\left|G|+\right|Z_{p}|$	3 $\left|G|+2\right|G_{T}\left|+svk+|\sigma \right|$

**Table 3:** Comparison of existing end-to-end encryption scheme.

Scheme	Confidentiality	Decentralization Authorization	Easy Deploy	Performance
[14]	No	No	Yes	-
[5]	Yes	No	Yes	Fast
[22]	Yes	No	No	-
[23]	Yes	No	No	-
[15]	Yes	No	Yes	Comparable to [20]
[20]	Yes	Yes	Yes	Slow (encrypt ≈ 42 ms, decrypt ≈ 62 ms)
Our scheme	Yes	Yes	Yes	Faster than [20] (encrypt ≈ 46 ms, decrypt ≈ 35 ms)

Note: - represents the performance of these schemes was not evaluated on our prototype.

## Data Availability

The data used to support the findings of this study are included in this article.

## Abbreviations

The following abbreviations are used in this manuscript:

IoT	Internet of Things
CPRE	conditional proxy re-encryption
MQTT	Message Queuing Telemetry Transport
AMQP	Advanced Message Queuing Protocol
TLS	Transport Layer Security
PRE	proxy re-encryption
CPS	Cyber–Physical Systems
WKD-IBE	identity-based encryption with wildcards
JPBC	The Java Pairing Based Cryptography Library
SDK	HiveMQ Extension SDK
AES	Advanced Encryption Standard
